# Urinary Bladder Patch Made with Decellularized Vein Scaffold Seeded with Adipose-Derived Mesenchymal Stem Cells: Model in Rabbits

**DOI:** 10.3390/biomedicines10112814

**Published:** 2022-11-04

**Authors:** Tadeu Ravazi Piovesana, Lenize da Silva Rodrigues, Ana Livia de Carvalho Bovolato, Diego Noé Rodríguez-Sánchez, Jaqueline Carvalho Rinaldi, Nilton José Santos, Julia Calvi Mori, Pedro Luiz Toledo de Arruda Lourenção, Lynn Birch, Matheus Bertanha

**Affiliations:** 1 Department of Surgery and Orthopedics, Botucatu Medical School, São Paulo State University-UNESP, Botucatu 18618-687, Brazil; 2 Applied Biotechnology Laboratory, Clinical Hospital of Botucatu Medical School, São Paulo State University-UNESP, Botucatu 18618-687, Brazil; 3 Postgraduate Program in Biosciences and Physiopathology, State University of Maringa, Maringá 87020-900, Brazil; 4 Department of Structural and Functional Biology, Institute of Bioscience of Botucatu, São Paulo State University—UNESP, Botucatu 18618-687, Brazil; 5 Department of Structural and Functional Biology, University of Campinas–UNICAMP, Campinas 13083-862, Brazil; 6 Department of Urology, College of Medicine, University of Illinois at Chicago, Chicago, IL 60612, USA

**Keywords:** mesenchymal stem cell, urinary bladder, urinary bladder diseases, tissue engineering, urologic surgical procedures

## Abstract

Background: To evaluate tissue regeneration of the urinary bladder after the implantation of a decellularized vein sown with autologous adipose-derived mesenchymal stem cells (ASC) on luminal surfaces. Methods: New Zealand rabbits (*n* = 10) were distributed in two groups: Group Bioscaffold alone (G1)-decellularized vena cava (1 cm^2^) was implanted, and Group Bioscaffold plus ACSs (G2)-decellularized vena cava (1 cm^2^) containing ASCs were implanted. ASCs were expanded, characterized, and maintained for one week in culture with a decellularized vein scaffold. The implants were performed under general anesthesia using a continuous suture pattern. Afterward, 21 d (day) specimens were collected and analyzed by hematoxylin and eosin (HE) histology and scanning electron microscopy (SEM). Results: The integrity of the urinary bladder was maintained in both groups. A superior regenerative process was observed in the G2 group, compared to the G1 group. We observed a greater urothelial epithelialization and maturity of the mucosa and submucosa fibroblasts. Furthermore, SEM demonstrated a notable amount of urothelial villus in the G2 group. Conclusion: Decellularized vena cava scaffolds were able to maintain the integrity of the urinary bladder in the proposed model. In addition, ASCs accelerated the regenerative process development, observed primarily by the new urothelial epithelization and the maturity of mucosa and submucosa fibroblasts.

## 1. Introduction

Several diseases can affect the urinary bladder and compromise its storage function, including congenital disorders, cancer, trauma, infection, chronic inflammation, and iatrogenic injuries [1]. In these clinical situations, reconstructive surgical procedures using non-urological autologous tissues become necessary [1]. In this sense, patches or neo-bladders, heterologous patches, and synthetic materials with autologous intestine segments that are more biocompatible are commonly used for bladder replacement or repair [2,3]. However, complications often occur using these tissues associated with recurrent infection, metabolic disorders, urolithiasis, perforation, excessive mucus production, obstructions, and neoplastic transformation [4]. In this context, further research is necessary to seek more efficient and lasting options.

Technological advances in tissue engineering with mesenchymal stem cells (MSC) have opened doors for new alternate substitutes based on the concept of personalized medicine [5,6]. Decellularized extracellular matrices help to support cell growth, tissue formation, and regeneration. Research with combinations of MSC and decellularized extracellular matrix (ECM) scaffolds in various tissue repairs has been extensively studied [7,8,9]. Among the advantages of this technology is the use of synthetic [5,6] or natural scaffolds derived from decellularized organic tissues. These are designed for maintaining physical structure, promoting the microenvironment for cell adhesion, and facilitating tissue integration and regeneration [10]. Decellularized ECM scaffolds consist primarily of ECM extracellular macromolecules, such as collagen, elastin, fibronectin, laminin, and matricellular proteins [11]. Furthermore, the physicochemical signals remain intact for regulating cell adhesion, proliferation, and differentiation, thus, providing a superior substrate for MSC seeding [7]. The scaffold produced through venous decellularization has biomechanical properties sufficient to resist the stresses of the urinary system and offers an extracellular matrix favorable to the cell population [12,13,14]. Tissue engineering research for the production and replacement of urinary bladder tissue has grown in recent decades; however, the clinical applications have not been completely established [15,16].

This present study aims to evaluate the biointegration and regeneration capacity induced in the urinary bladder in a rabbit model by comparing urothelial reconstitution of the bladder using decellularized vena cava versus vena cava sowed with autologous adipose-derived mesenchymal stem cells (ASC), based on a previously established vein decellularization model [7,11,17,18,19,20].

We hypothesize venous scaffolds populated with autologous stem cells provide a favorable environment for the maintenance of the structure and regeneration of the urothelium.

## 2. Materials and Methods

### 2.1. Animals Housing Conditions, and Ethics

Fifteen 3-month-old female New Zealand rabbits, each weighing 2.5 kg, were housed under controlled conditions and fed a standard pellet diet with water ad libitum. 

All animals were handled in accordance with NIH Guidelines for the Care and Use of Laboratory Animals (NIH 1996) [21] with ethical approval from the institutional Ethics Committee of Animal Use (CEUA protocol approval #1298/2019).

### 2.2. Experimental Groups

Five rabbits each were placed into two experimental groups. Group Bioscaffold alone, (G1), underwent surgical implantation of a 1 cm^2^ decellularized vena cava segment (*n* = 5). Group Bioscaffold seeded with ASCs, (G2), underwent surgical implantation of a 1 cm^2^ decellularized vena cava segment seeded internally with autologous adipose-derived mesenchymal stem cells (ASC) (*n* = 5). The five remaining rabbits were used to obtain the scaffold bank as described below.

### 2.3. Anesthetic Procedures

A qualified veterinarian assisted with the anesthetic procedure. Prior to surgical incision for autologous adipose tissue collection, the rabbits were premedicated intramuscularly with a combination of ketamine 10 mg/kg (Dopalen^®^, 100 mg/mL, CEVA, Paulínia, SP, Brazil), xylazine hydrochloride 3 mg/kg (Anasedan^®^ 2%, 20 mg/mL, CEVA, Paulínia, SP, Brazil), and acepromazine 0.1 mg/kg (Apromazin^®^ 0.2%, 200 mg/mL, Barueri, SP, Brazil). Additionally, Lidocaine 7 mg/kg (Xylocaine 1% 20 mg/mL, Cristália, Butantã, SP, Brazil), a topical anesthetic, was administered at the projected site of the surgical incision.

During laparotomy, the rabbits were maintained under general inhalation anesthesia with 3–4% isoflurane (Isoforine^®^, Cristália, Itapira, SP, Brazil) diluted with 100% oxygen. An intravenous 0.9% saline solution was administered for hydration. Upon laparotomy completion, the rabbits were observed for 21 days and then euthanized with a thiopental lethal overdose (Thiopentax, 120 mg/kg, Cristália, Itapira, SP, Brazil).

### 2.4. Tissue Collection

#### 2.4.1. IVC Decellularization

The infrarenal inferior vena cava (IVC) were obtained from five rabbits to compose the scaffold bank. One rabbit was selected as the in natura bladder donor used as a normality control. The IVCs were fragmented into 1 cm length segments and processed with the standardized decellularization protocol using 1% sodium dodecyl sulfate (SDS, Sigma-Aldrich, Santo André, SP, Brazil) with a 2 h, 37 °C incubation on a shaker (Supplemental File S1). The decellularization process aims to remove all cellular and nuclear material, including antigens, while preserving the structural and mechanical integrity and chemical properties of the extracellular matrix [12,13]. The decellularized fragments were maintained in a sterile solution containing antibiotic ciprofloxacin (Ciprofloxacin 10 mg/mL, Bayer, Paulina, SP, Brazil) and antifungal amphotericin B (Amphotericin B 20 mg/mL, Geolab, Anápolis, GO, Brazil) at 4 °C for 7 days.

#### 2.4.2. ASC Collection and Characterization

The autologous ASCs for the Group Bioscaffold plus ASCs (G2) were isolated from adipose tissue samples from the five rabbits of G2 and seeded into the scaffold for subsequent implantation in an autologous way. A 1.5 cm interscapular transverse skin incision was created to obtain 1 g of fat tissue from this region. The wound was sutured with simple Nylon 4–0 stitches (Ethicon™, Somerville, NJ, USA). The adipose tissue samples were processed, according to the enzymatic digestion protocol [22]. Briefly, a solution of collagenase type I (Invitrogen^TM^, Thermo-Fisher, Waltham, MA, USA) at 4 mg/g adipose tissue was prepared in 2 mL of HEPES. The adipose tissue was incubated in the collagenase type I solution for 15 h at 37 °C to complete the enzymatic digestion process. Finally, the fraction of lymphomononuclear cells that contain ASCs was obtained as 5 × 10^4^ cells/g of adipose tissue. The cells were seeded in culture flasks and expanded for 3 weeks in a culture medium containing Dulbecco’s Modified Eagle Medium (DMEM, Gibco, Thermo-Fisher, Waltham, MA, USA) supplemented with 10% fetal bovine serum (DMEM/FBS) (Fetal bovine serum, Gibco, Thermo-Fisher, Waltham, MA, USA) and placed in an incubator at 37 °C with 5% CO_2_. A sample of 1 × 10^6^ ASCs from a second replicate of cultures from each animal was subjected to phenotypic characterization by flow cytometry for cell surface markers CD90 and CD44 or the absence of CD45 and CD11b (all from BD Biosciences, San Jose, CA, USA) (Supplemental File S2). Multipotent characteristics were confirmed by differentiation tri-lineage capacity (Supplemental File S3).

### 2.5. Application of ASCs in Decellularized Vein Scaffolding

The IVC scaffolds were sectioned longitudinally to obtain a 0.8 × 1 cm rectangular fragment and fixed with the luminal side facing upward in the well bottom of a 6-well non-adherent culture plate using a drop of Puramatrix (Corning, Tewksbury, MA, USA). For the Group Bioscaffold plus ASCs (G2), a 5 µL solution of Puramatrix mixed with 1 × 10^6^ ASCs was prepared and applied directly onto the IVC scaffold. Gelation was stimulated with 10 µL of DMEM/FBS culture medium (All from Gibco, Thermo-Fisher, Waltham, MA, USA) and incubated for 30 min. For both groups, the well was supplemented with 2 mL of DMEM/FBS culture medium and maintained in culture for 1 week at 37 °C. The medium was changed every two days.

### 2.6. Surgical Procedure for Bladder Scaffold Implantation

For both the G1 and G2 groups, a small 3 cm suprapubic longitudinal laparotomy was performed to access the abdominal cavity. The urinary bladder was identified and exposed for graft implantation. A rectangular section of approximately 0.8 cm × 1.0 cm from the urinary bladder wall on the anterior and apical face was excised for implantation of the patch, consisting either of a decellularized vein only (G1 group) or a decellularized vein seeded with ASCs (G2 group). The patch was fixed with continuous Vicryl 7.0 sutures (Ethicon^TM^, Somerville, NJ, USA). The abdominal wall was sutured closed in 2 planes. The animals were housed in a vivarium and monitored daily for 21 days. Antimicrobial prophylaxis was dispensed IM with enrofloxacin (10 mg/kg, Baytril^®^, Bayer, Paulina, SP, Brazil) for 7 d, and if necessary additional analgesia with tramadol hydrochloride (5 mg/kg, Tramal^®^, Aachen, Germany) was administered.

At 21 days post-surgery, the animals were euthanized, and the implanted tissue was surgically removed. The collected specimens were divided in half and fixed in either a 10% buffered solution formalin for histological analysis or in glutaraldehyde for scanning electron microscopy.

### 2.7. Confirmation of Cell-Seeding

Bioscaffolds seeded with ASCs and cultured for 1 week were evaluated for the presence of cells on the surface. A 1 mm cross-section was taken from each bioscaffold to perform frozen section immunofluorescence nuclear labeling with 4′,6′-diamino-2-phenyl-indole (DAPI, Sigma-Aldrich, Santo André, SP, Brazil)) to observe the cells’ nuclei on the surface.

### 2.8. Histology

Two slides each with four (5 µm thick) paraffin sections were processed for each animal. Slides were stained with hematoxylin-eosin (HE) and picrosirius red (PS), respectively. For histological analysis, HE slides were scanned and digitized using a Pannoramic MIDI (3DHistech, Budapest, Hungary) with a 3DHistech Pannoramic Viewer^®^ software (version 1.15.4.43061 SP1, 25 July 2014). Quantitative analysis from digital images of PS-stained slides was performed to calculate the area occupied by the deposition of collagen in the bladder wall. The images were captured using a Leica DMC2900 digital video camera (Leica Microsystems, Germany) coupled to a Leica DM2500 light microscope using a Leica Qwin image analysis software version 3.1. The microscope was equipped with a light circular polarizer composed of a simple polarizer and a filter analyzer. This filter was aligned so the background in the field of view would be as dark as possible.

### 2.9. Morphometry

#### 2.9.1. Inflammatory and Repair Evaluation Score

Histological sections stained with HE were analyzed blindly by assigning the following repair scores: (a) inflammatory process severity [23], (b) predominant inflammatory cell types [24], (c) epithelial repair [25], and (d) connective tissue repair [26], according to Table 1.

#### 2.9.2. Collagen Fiber Analysis and Quantification

The paraffin sections (5 mm) were stained by picrosirius red (PS) for collagen fibers (subtypes I and III) measurement. Collagen type III (fibrillar-immature) was identified by green birefringence, whereas collagen type I (dense-mature) was identified by yellow-red birefringence. Ten unique fields (200× magnification) from two different sections of five samples were evaluated blindly per group. Image analysis was made using ImageJ v1.49 software (National Institute of Health, Bethesda, MD, USA). The collagen types I and III were calculated using the “threshold” function and “color segmentation” plugin (EPFL, Lausanne, Switzerland) (Supplemental Figure S2) and were expressed as a fraction area (%) of each image. This ImageJ plugin allows a color image or a stack of colors to be segmented by pixel clustering (2048 × 1536 pixels). The cluster was defined manually by the user through the interface. Blind analysis was performed by an independent evaluator to score for the presence of collagen I, III, and total.

In addition, 5 mm paraffin sections were treated with collagen fiber subtype I and III primary antibodies (Dako™, Glostrup, Denmark) and evaluated by immunohistochemical analysis.

#### 2.9.3. Scanning Electron Microscopy (SEM)

Glutaraldehyde-fixed samples were prepared in 0.5% osmium tetroxide and dehydrated in alcohol. A Quanta 200 scanning electron microscope (Fei Company, Hillsboro, OR, USA) was used to image and analyze qualitatively the samples, in comparison to the in natura bladder. To metalize the samples for higher quality images, 24 k pure gold powder was used (Supplemental File S4).

### 2.10. Statistical Analysis

Continuous numerical data are expressed as median (first and third quartile), according to the nonparametric distribution, previously determined by the Kolmogorov–Smirnov normality test. Comparisons between these variables were made using the Mann–Whitney test. The level of significance considered was 5% and all analyses were performed using SPSS 22.0 software.

## 3. Results

### 3.1. Confirmation of Cell-Seeding

**Figure 1:**
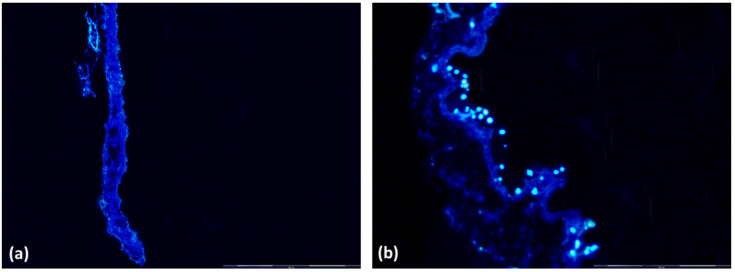
Immunofluorescence of bioscaffolds and nuclear labeling with DAPI. (**a**) Group 1 Bioscaffold alone (G1) without nuclei; (**b**) Group 2 Bioscaffold seeded with ASCs (G2) with nuclei labeling in blue. Magnification = 400×.

Bioscaffold samples seeded with ASCs (G2) showed an adequate cell population before implantation into the bladder by DAPI nuclear-labeled immunofluorescence analysis of frozen sections, compared to the cell-free bioscaffold (G1) (Figure 1).

### 3.2. Tissues Collection

#### 3.2.1. IVC Decellularization

HE stained images validated successful inferior vena cava decellularization (Supplemental Figure S1).

#### 3.2.2. ASC Characterization

ASCs isolated from each rabbit were analyzed for phenotypic characterization by flow cytometry. MSC cell surface markers CD44 and CD90 displayed positive event peaks, while hematopoietic and immune cell markers CD45 and CD11b, respectively, were negative. Supplemental Figure S3 shows representative plots. MSC’s tri-lineage differentiation efficiency was verified by examining its ability to differentiate into mature adipocytes, osteoblasts, and chondrocytes (Supplemental Figure S4). Specific dyes for each cell type revealed large areas of differentiated cells.

### 3.3. Macroscopy

A good tissue integration in all animals of G1 was observed. However, severe external fibrosis was observed in two animals with regenerative tissue in the internal wound. Calculus adhered in the urinary bladder close to the patch implantation in two rabbits and there was no formation of fistulas around the wound. On the other hand, good tissue integration was observed in the rabbits of the G2 group with mild external fibrosis and a good presence of tissue regenerated in the internal wound. 

**Figure 2:**
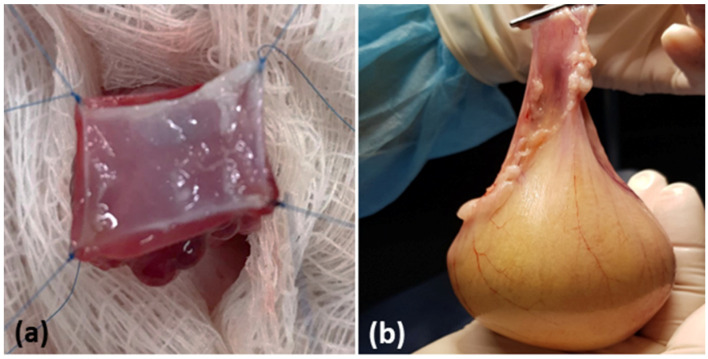

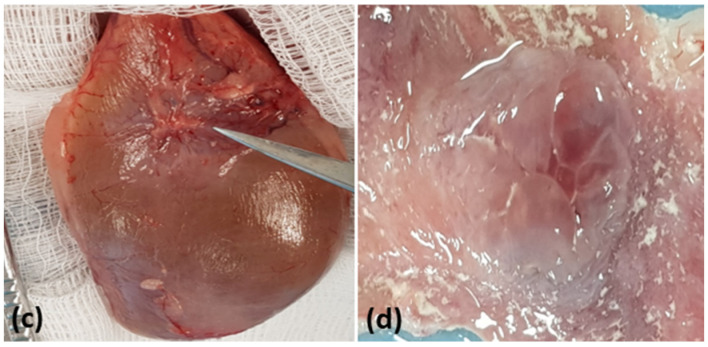
Macroscopic aspects of the urinary bladder. (**a**) Surgical implantation of the scaffold in the total wall. (**b**) Euthanasia and surgical removal of the urinary bladder for analysis. (**c**) The tip of the scissors indicates the scaffold implant point. (**d**) Macroscopy of the scaffold implanted on the luminal surface of the bladder.

There were no significant macroscopic differences between groups (Figure 2).

### 3.4. Microscopy

**Figure 3:**
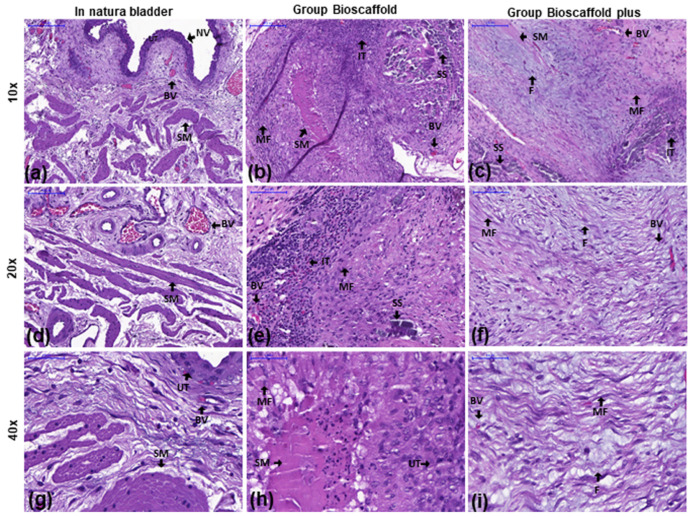
Inflammatory infiltration analysis of the urinary bladder by hematoxylin and eosin stains at three magnifications. Groups: In natura (**a**,**d**,**g**): Urothelium with normal structure and normal-looking villi (NV). Bioscaffold alone (**b**,**e**,**h**): Infiltrating inflammatory tissue (IT) and immature myofibroblasts (MF) were observed in the submucosa near the wound. Bioscaffold group with ASCs (**c**,**f**,**i**): Urothelium with normal structure and infiltrating inflammatory tissue (IT). Mature fibroblasts (F) were observed in the submucosa close to the wound. Blood vessels (BV) were observed in the region equivalent to the vesical mucosa/submucosa transition. Key: blood vessel (BV), smooth muscle (SM), myofibroblasts (MF), urothelium (UT), infiltrating tissue (IT, with macrophages, lymphocytes, eosinophils, plasmocytes, and neutrophils), surgery stitches (SS), normal-looking villi (NV), mature fibroblasts (F). Magnification: Top row: 10×; Middle row: 20×; Bottom row: 40×.

Figure 3a,d,g show the normal structure of the urinary bladder (urothelium with normal structure and normal-appearing villi (NV)).

In the G1, inflammatory infiltration was observed characterized by the presence of macrophages, lymphocytes, eosinophils, plasmocytes, and neutrophils. Immature myofibroblasts (MF) shown with arrows were observed in the submucosa close to the wound (Figure 3b,e,h).

In the G2, inflammatory infiltration was observed composed of several cell types, including macrophages, lymphocytes, eosinophils, plasmocytes, and neutrophils. Moreover, mature fibroblasts were observed in the submucosa close to the wound. Interestingly, a greater formation of blood vessels (BV) was observed in the region equivalent to the vesical mucosa/submucosa transition (Figure 3c,f,i).

### 3.5. Morphological Score

Slides stained in HE showed statistical differences in severity scores of the inflammatory process, the types of inflammatory cells, epithelial repair, and the tissue repair scores favorable to G2, as shown in Table 2.

### 3.6. Collagen in Bladder Extracellular Matrix

**Figure 4:**
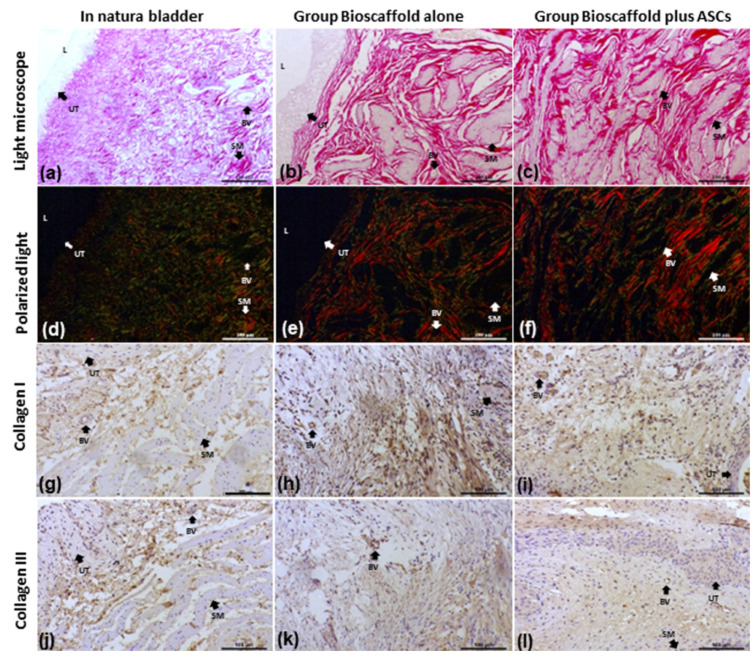
Collagen I and III analysis by several methods. Groups: In Natura (**a**,**d**,**g**,**j**); Bioscaffold alone (**b**,**e**,**h**,**k**); Bioscaffold group with ASCs (**c**,**f**,**i**,**l**). Analysis Methods: (**a**–**c**) picrosirius red-stained slides for collagen I and III fiber measurements; (**d**–**f**) Polarized light exposed slides identified collagen type III (fibrillar-immature) by green birefringence and collagen type I (dense-mature) by yellow-red birefringence; (**g**–**i**) Immunohistochemistry staining for Collagen I; (**j**–**l**) Immunohistochemistry staining for Collagen III. Key: blood vessel (BV), smooth muscle (SM), urothelium (UT), lumen (L). Magnification: Top row:100×; Middle row: 1500×; Bottom row 6000×.

Under polarized light, a red-orange and green birefringence spectrum were observed, suggesting the presence of collagen types I and III that were distributed homogeneously in the urinary bladder wall and close to the implant. Collagen fibers type I and III quantifications are presented in Table 3. Collagen type I was higher in samples from G2, compared to the G1 group (*p* < 0.0001). Collagen type III was not significantly different between groups (*p* = 0.1068) (Figure 4).

In addition, collagen fibers I and III by immunohistochemical analysis revealed an equal distribution in the transitional epithelium, the lamina propria of the mucosa, and between the muscle fibers in all groups (Figure 4).

### 3.7. Scanning Electron Microscopy (SEM)

**Figure 5:**
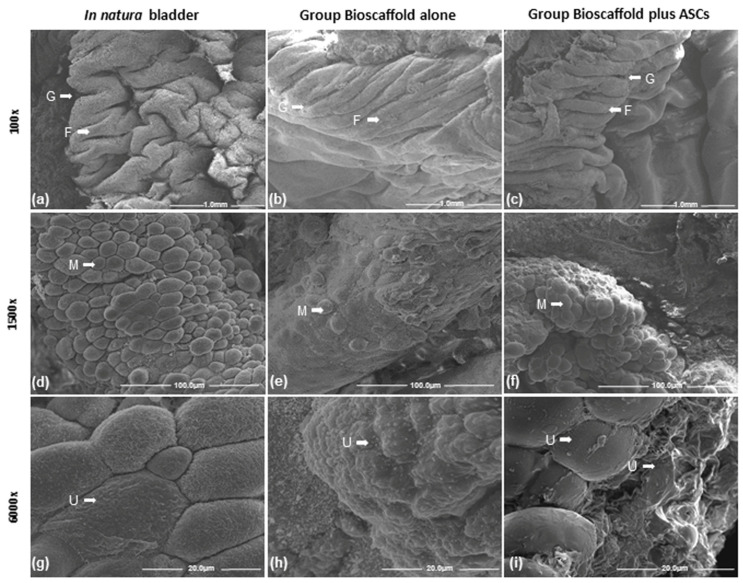
Scanning electron microscopy of the urinary bladder. Group comparisons of urinary bladder cell characteristics of folds and grooves (fissures) at three magnifications. Groups: In natura (**a**,**d**,**g**); Bioscaffold alone (**b**,**e**,**h**); Bioscaffold group with ASCs (**c**,**f**,**i**). Key: microvilli (M), folds (F), grooves (G), urothelium (UT). Magnification: Top row: 100×; Middle row: 1500×; Bottom row: 6000×.

Images of the urinary bladder surface were captured by SEM for ultrastructural analysis. At 100×, the lowest magnification showed several folds and grooves (fissures) of the urinary bladder (Figure 5a–c). At 1500× magnification, the surface structure showed a wide range of bubbles or microvilli coverage on the transition epithelium (urothelium) (Figure 5d–f). At a 6000× higher magnification, SEM images showed more precise details of the bladder surface bubbles or microvilli on the transitional epithelium (urothelium) (Figure 5g–i). The urothelium of the G2 was similar to the in natura urinary bladder tissue. The superficial cells of the urothelium in G2 were smaller and slightly more rounded than the same cells in natura urinary bladder tissue but also preserved cell-to-cell contact. Differently, the G1 Group Bioscaffold alone presented more flattened cells, which were smaller and in fewer numbers, compared with the G2 Group Bioscaffold plus ASCs and the in natura urinary bladder tissue.

## 4. Discussion

Natural materials, synthetic polymers, and approaches based on decellularization have been developed to obtain biocompatible materials for tissue grafts or complete im-plantation of the organ [27,28,29,30,31]. Biomaterial grafts become functionalized when using autologous stem cells due to the proliferative and self-renewal capacity of stem cells associated with anti-inflammatory, immunomodulatory, and pro-regenerative mechanisms [32,33]. Current studies utilizing the application of mesenchymal stem cells in decellularized biological structures to regenerate other various tissues, such as bone, cartilage, kidney, blood vessels, and heart, have demonstrated positive effects, highlighting the organized regenerative potential and inhibiting the disordered inflammatory process [9,34,35,36,37]. Acellular matrices used for vesical augmentation to produce a homogeneous extracellular matrix membrane can be prepared from the intestine, stomach, bladder, and other tissue types by either mechanical or chemical manipulation. Sutherland et al. [27] have shown epithelialization and smooth muscle regeneration matured into normal-sized bundles and vascularization within 26 weeks in decellularized stomach matrices implanted in the urinary bladder of rats. Vaught et al. [28], obtained good detrusor muscle regeneration through the implantation of porcine small bowel submucosa grafts, resulting in a functional capacity of contractility in a rat urinary bladder augmentation model. In our present study, we obtained a good integration of the decellularized vein patch and uroepithelial renovation. There were fewer inflammatory responses detected in group 2 (bioscaffold with ASC) compared with group 1 (bioscaffold alone) at 3 weeks after the implantation on the urinary bladder.

For our studies, the parameters evaluated included the observation of epithelization and calcification or the formation of bladder stones among the G1 and G2 groups. Factors that may have positively influenced urothelization may be related to the size of the im-planted decellularized vein fragment. The 1 cm^2^ patch favored the urothelial growth from the edges to the center, but, even so, the group that received ASCs had an improved quality of urothelization, compared with Group 1 (bioscaffold alone). However, it is not clear what the role of ASC is in the urothelial restocking of the implanted segment. Additional studies analyzing the role of ASC during urothelization will need further investigation. Bladder stones were observed even when the graft remained in direct contact with urine. A possible explanation could be the bladder environment’s alkaline pH causing the precipitation of salts in the presence of foreign bodies [37].

Lepper et al. (2002) [3] evaluated the presence of bladder tissue regeneration after 3 months of augmentation with anionic collagen membrane (ACM) in the rabbit model. The researchers found that the effectiveness of bladder augmentation capacity was not different with or without urothelium preservation. In our study, we observed a significant presence of an intense inflammatory process in the Group Bioscaffold alone (G1), when compared with the Group Bioscaffold plus ASCs (G2).

Studies using acellular collagen membrane grafting to perform bladder augmentation showed normal cellular organization of epithelium, submucosa, and muscle formation in the long-term enlarged bladder [38]. Other authors concluded that this type of graft must be completely absorbed without tissue rejection, avoiding adherences and mutagenic alterations after implantation for bladder tissue regeneration [39,40]. Our results corroborate this literature, where none of the animals rejected their graft implants.

Scaffolds with biomechanical properties utilizing an extracellular collagen matrix for supporting cell growth and survival have proven to be an ideal model for engineering bladder tissue. In addition, there is a need for sufficiently neovascularized constructs that support and provide nutrients, oxygen, and viability of cells [41]. The vena cava ECM structure is a promising framework for stimulating tissue regeneration that can be used as a scaffold to support MSC in tissues other than the urinary bladder [42,43].

## 5. Conclusions

Decellularized rabbit vena cava scaffold maintained the urinary bladder integrity at 21 days after implantation. After recellularization with ASCs, a superior regenerative process was developed, characterized by urothelial epithelialization, the maturity of the mucosa, and the submucosal population by fibroblasts. These findings could represent a new direction for the development of tissue engineering applied to the urinary bladder.

## Figures and Tables

**Table 1:** Post-Surgical Repair Scores.

Inflammation Scores *	(1) Without inflammatory cells
	(2) Mild inflammatory infiltration (cell infiltration)
	(3) Moderate inflammatory infiltration (cell infiltration + vascular congestion)
	(4) Severe inflammatory infiltration (cell infiltration + vascular congestion + calcification/necrosis)
Inflammatory Cell Types ^¥^	(1) Predominance of polymorphonuclear cells
	(2) Predominance of mononuclear cells
	(3) Mono and polymorphonuclear inflammatory cells
Epithelial Repair Scores ^§^	(1) Complete wall reepithelialization
	(2) Partial reepithelialization (with or without intraepithelial inflammatory cells)
	(3) Without reepithelialization
Connective Tissue Repair Scores ^×^	(0) Absence of fibrosis
	(1) Mild fibrosis (fibers interspersed with non-fibrillar ECM)
	(2) Moderate/severe fibrosis (thick fibers, not individualized with little or no non-fibrillar ECM)

Modified from: * McClanahan, SB 1991 [23]; ^¥^ Ter Woort, F 2018 [24]; ^§^ Nogueira, MP 2008 [25]; ^×^ Queiroz AM et al., 2011 [26].

**Table 2:** Analysis of post-surgical repair scores.

Score	Group#1	Group#2	*p*
Inflammatory Scores *	*n* = 180	*n* = 150	0.0019
	1 (1–2)	1 (1–2)	
Inflammatory Cell Types ^¥^	*n* = 184	*n* = 149	0.0019
	0 (0–2)	0 (0–2)	
Epithelial Repair Scores ^§^	*n* = 36	*n* = 30	0.0129
	1 (1–2)	1 (1–1)	
Connective Tissue Repair Scores ^×^	*n* = 36	*n* = 30	0.0088
	1 (1–2)	0.5 (0–1)	

Modified from: * McClanahan, SB 1991 [23]; ^¥^ Ter Woort, F 2018 [24]; ^§^ Nogueira, MP 2008 [25]; ^×^ Queiroz AM et al., 2011 [26].

**Table 3:** Qualitative Analysis of Collagen Fibers I and III.

Score	Group#1	Group#2	*p*
Collagen I	*n* = 83	*n* = 62	<0.0001
	15.97 (12.722–18.534)	19.04 (17.726–22.311)	
Collagen III	*n* = 83	*n* = 62	0.1068
	7.62 (6.596–8.734)	7.76 (6.996–9.068)	
Total Collagen (I + III)	*n* = 83	*n* = 62	<0.0001
	24.09 (20.621–26.744)	28.19 (25.691–30.275)	

## Data Availability

All survey data can be requested from the corresponding author.

## Supplementary Materials

The following supporting information can be downloaded at: https://www.mdpi.com/article/10.3390/biomedicines10112814/s1, supplementary file [44,45].
