# Supplementary material for: Urinary Bladder Patch Made with Decellularized Vein Scaffold Seeded with Adipose-Derived Mesenchymal Stem Cells: Model in Rabbits
Source: Biomedicines. 2022 Nov 4;10(11):2814. doi: 10.3390/biomedicines10112814 (PMC9687924; doi:10.3390/biomedicines10112814)
Supplement: Supplementary file 1 [file biomedicines-10-02814-s001.zip › biomedicines-1980910-supplementary.pdf]

Supplemental Files

Index

Supplemental File S1 - Vein decellularization protocol

Supplemental Figure S1. Evidence of inferior vena cava decellularization

Supplemental Figure S2. How to access Image-Pro Plus for collagen quantification

Supplemental File S2 - Phenotypic characterization of ASC by flow cytometry analysis

Supplemental Figure S3. Phenotypic characterization by flow cytometry for cell surface markers of rabbit #1.

Supplemental File S3 -Tri-linage Differentiation

Supplemental Figure S4. Tri-lineage Differentiation.

Supplemental File S4 - Sample processing protocol for SEM

Supplemental File S1 - Vein decellularization protocol

Vein decellularization protocol

Material

- Conical tube
- Injection water
- Sodium duodecyl sulfate (SDS) 1%
- Clean and fragmented sample

Solution preparation Sodium duodecyl sulfate (SDS) 1%: 200mL of ultrafiltered H20 + 2.5g SDS (molecular weight 288.38, concentration 0.35 M, Sigma Aldrich, San Luis, Missouri, USA)
Ps. Each conical tube must be filled with 25 mL of the 1% SDS solution.

Procedure

In a laminar flow chamber, sterile environment, the samples (veins) must go through the cleaning procedure to ensure that there are no remains of blood and fat compound, then separated into fragments according to the study protocol. The fragmented samples will be stored at -80ºC until the moment of decellularization.

Before the decellularization process, the fragments will be thawed at room temperature, washed with saline solution, and stored 5 samples per conical tube containing 25mL of 1% SDS solution, seal the lid with parafilm to prevent leakage. Place the tubes in a horizontal position on the shaker tray and fix them with tape, keep stirring at 1200rpm for 2 hours at 37ºC (Shaker News Brunswick Scientific® with the controlled temperature at 37ºC). The fragments went through a cycle of 3 washes with sterile saline solution and were conditioned and preserved in a refrigerator at 4ºC in a sterile solution containing antibiotic and antifungal until the moment of use in culture.

Observations: Use mask for handling SDS, mucosa irritant substance.

[1] Bertanha M, Sobreira ML, Bovolato ALC, Rinaldi JC, Reis PP, Moroz A, Moraes LN, Deffune E. Ultrastructural analysis and residual DNA evaluation of rabbit vein scaffold. Acta Cir Bras. 2017 Sep;32(9):706-711.

[2] Bertanha M, Moroz A, Jaldin RG, Silva RA, Rinaldi JC, Golim MA, Felisbino SL, Domingues MA, Sobreira ML, Reis PP, Deffune E. Morphofunctional characterization of decellularized vena cava as tissue engineering scaffolds. Exp Cell Res. 2014 Aug 1;326(1):103-11.

[3] Rodrigues LDS, Bovolato ALC, Silva BE, Chizzolini LV, Cruz BLD, Moraes MPT, Lourenção PLTA, Bertanha M. Quantification of adhesion of mesenchymal stem cells spread on decellularized vein scaffold. Acta Cir Bras. 2021 Nov 5;36(10):e361001.

Supplemental Figure S1. Evidence of inferior vena cava decellularization

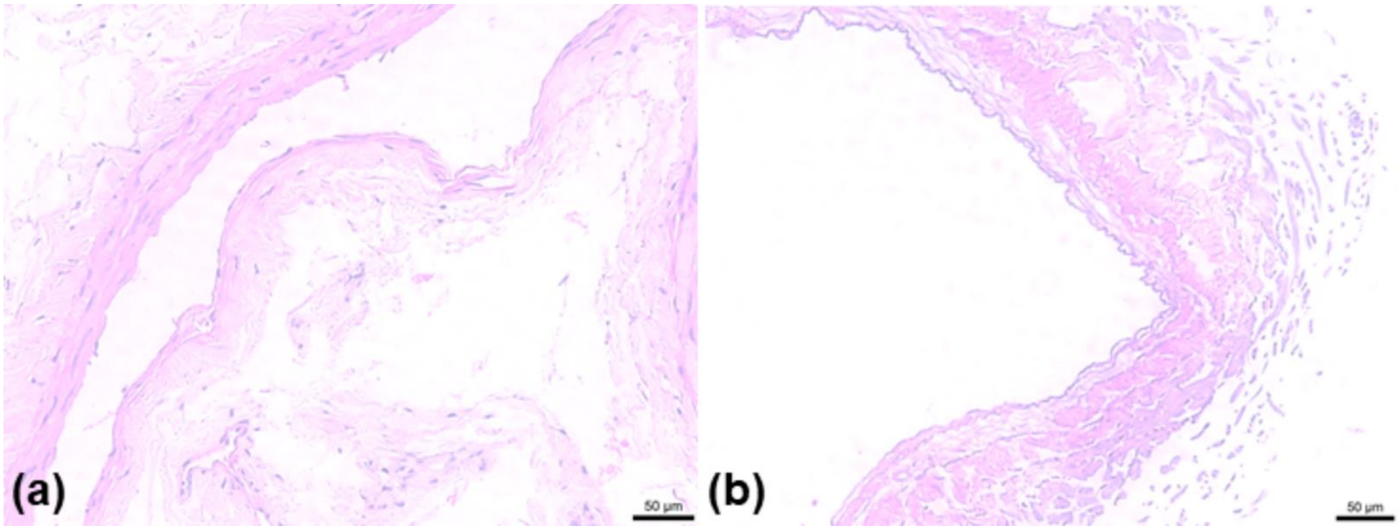

Figure S1. Evidence of inferior vena cava decellularization. H&E staining of **(a)** Vein in natura; **(b)** Successful decellularization of the inferior vena cava. Magnification: 50μm

Supplemental Figure S2

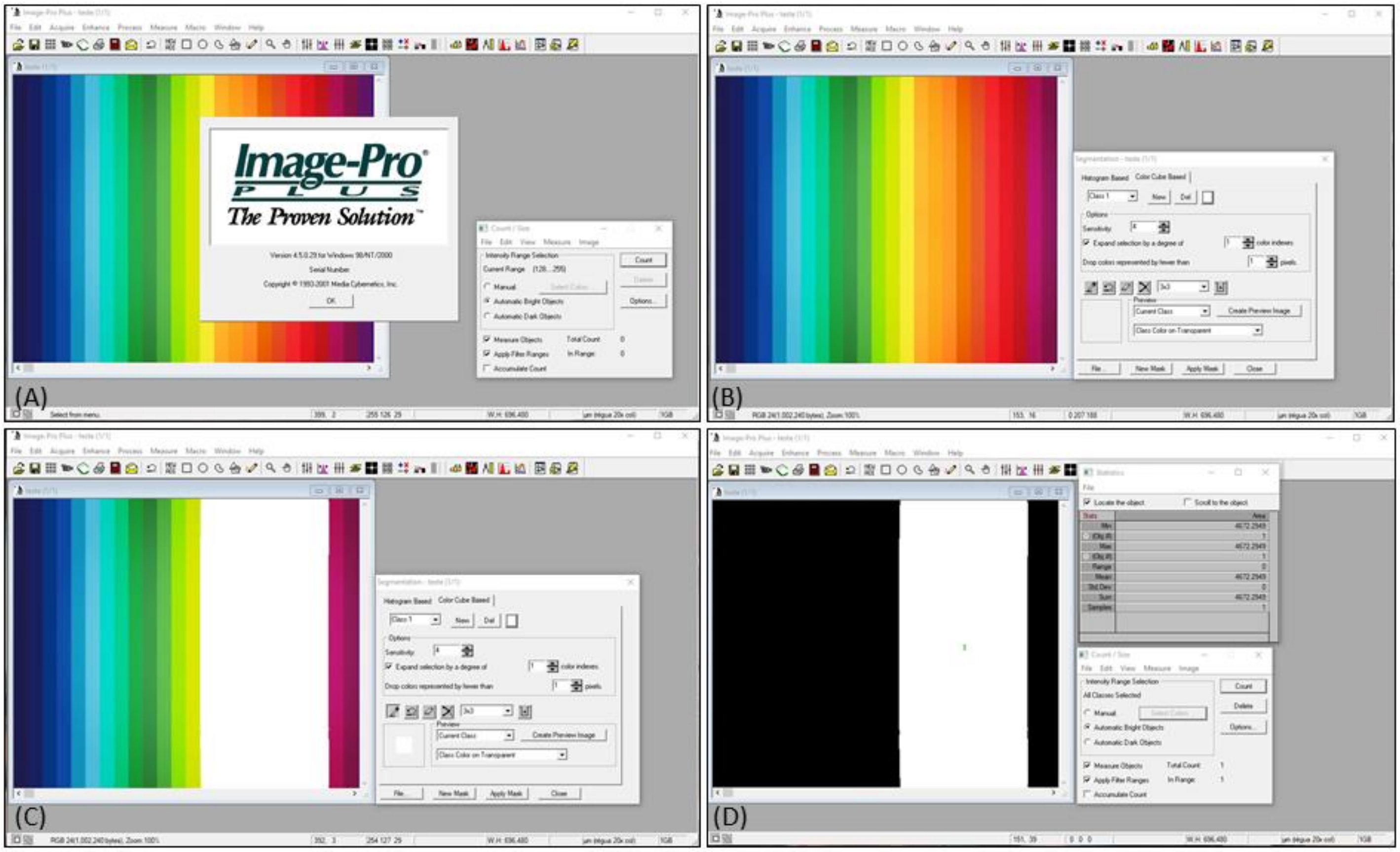

Supplemental Figure S2. Access Image-Pro Plus -> include the photomicrograph -> place the appropriate ruler -> measure -> count/size -> "Manual:" option -> "Select Colors ..." option -> Segmentation -> Choose a color that you don't have in your image for contrast -> Select the "eyedropper" button -> Color in the image what you want to count -> Finished, "apply mask" -> Close -> "Automatic Bright Objects" option -> Button "count" -> "View" option -> "statistics" option -> copy the value.

Supplemental File S2 - Phenotypic characterization of ASC by flow cytometry analysis

Flow cytometry analysis of the phenotypic characterization of cell surface markers on adipose-derived mesenchymal stem cells (ASC). The expressions of positive (CD90-Percp and CD-44 FITC) and negative (CD45-PE and CD11b-APC) cell surface marker distribution were evaluated using the Facscalibur BD™ flow cytometer using the Cell Quest™ software. The control reagent used was what was available on the market, an anti-MSC from rats with cross-reactivity with rabbits. Samples prepared for the assay contained $2x10^5$ cells per tube. Antibodies were purchased from BD Biosciences®.

Supplemental Figure S3

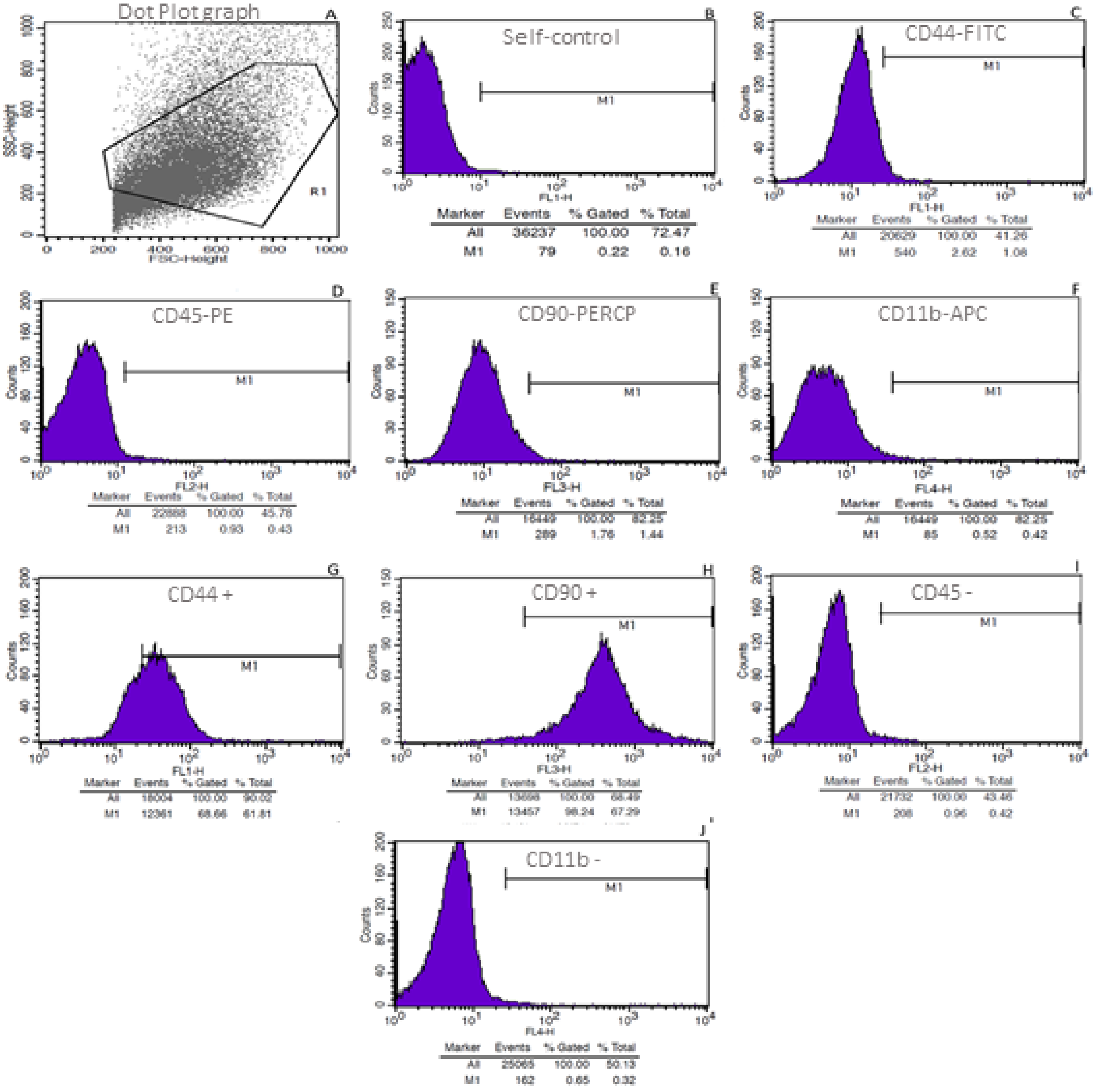

Supplemental Figure S3. Representative phenotypic characterization by flow cytometry for cell surface markers from one rabbit ASC. Dot Plot graph **A** represents the distribution of the cell population. Immature cells are large-sized and have less granularity, while mature cells are small-sized and have greater granularity. This would correspond to the second peak found in the histogram distribution by flow cytometry [1-2]. Histogram **B** shows self-control for the evaluation of cell autofluorescence. Histograms **C, D, E,** and **F** are of iso-controls used to evaluate and calibrate the equipment with the fluorescence of the respective antibodies/fluorochromes CD44-FITC, CD45-PE, CD90-PERCP, and CD11b-APC, respectively. Histograms **G** and **H** show MSC markers CD44 and CD90, respectively, with positive peaks. Histograms **I** and **J** show hemopoietic and immune markers CD45 and CD11b, respectively, with no peaks (negative). All markers used correspond to those described in the literature.

[1] Docheva D, Padula D, Popov C, Mutschler W, Clausen-Schaumann H, Schieker M. Researching into the cellular shape, volume and elasticity of mesenchymal stem cells, osteoblasts and osteosarcoma cells by atomic force microscopy. J. Cell Mol. Med. 2008;12: 537-552.

[2] Gugjoo MB, Kinjavdekar AP, Aithal HP, Ansari MM, Pawde AM, Sharma GT. Isolation, Culture, and Characterization of New Zealand White Rabbit Mesenchymal Stem Cells Derived from Bone Marrow. X Asian Journal of Animal and Veterinary Advances. 2015;10: 537-548.

Supplemental File S3 -Tri-linage Differentiation of cartilage, fat, and bone.

Characterization by differentiation with inducing or "tri-lineage" is defined as having the function of attesting to the potential for differentiation of MSCs. At 80% confluency, cells were introduced to three specific inducing media: Stempro-adipogen™, Stempro-chondro™, and Stempro-osteogen™ (Life Technologies®) and were used according to the manufacturer's instructions. In 24-well plates, the MSCs were cultured in the presence of an inducing medium for a specific time, stained with an appropriate cell dye, and microscope images recorded. MSCs cultured for 21 days to induce chondrogenic differentiation were stained with Alcian Blue® to visualize collagen depositions; MSCs cultured for 14 days to induce adipogenic differentiation were stained with Oil Red® to visualize lipid droplets, and MSCs cultured for 14 days to induce osteogenic differentiation were subjected to an Alizarin Red® dye for staining calcium deposits. Media was changed twice a week.

Supplemental Figure S4

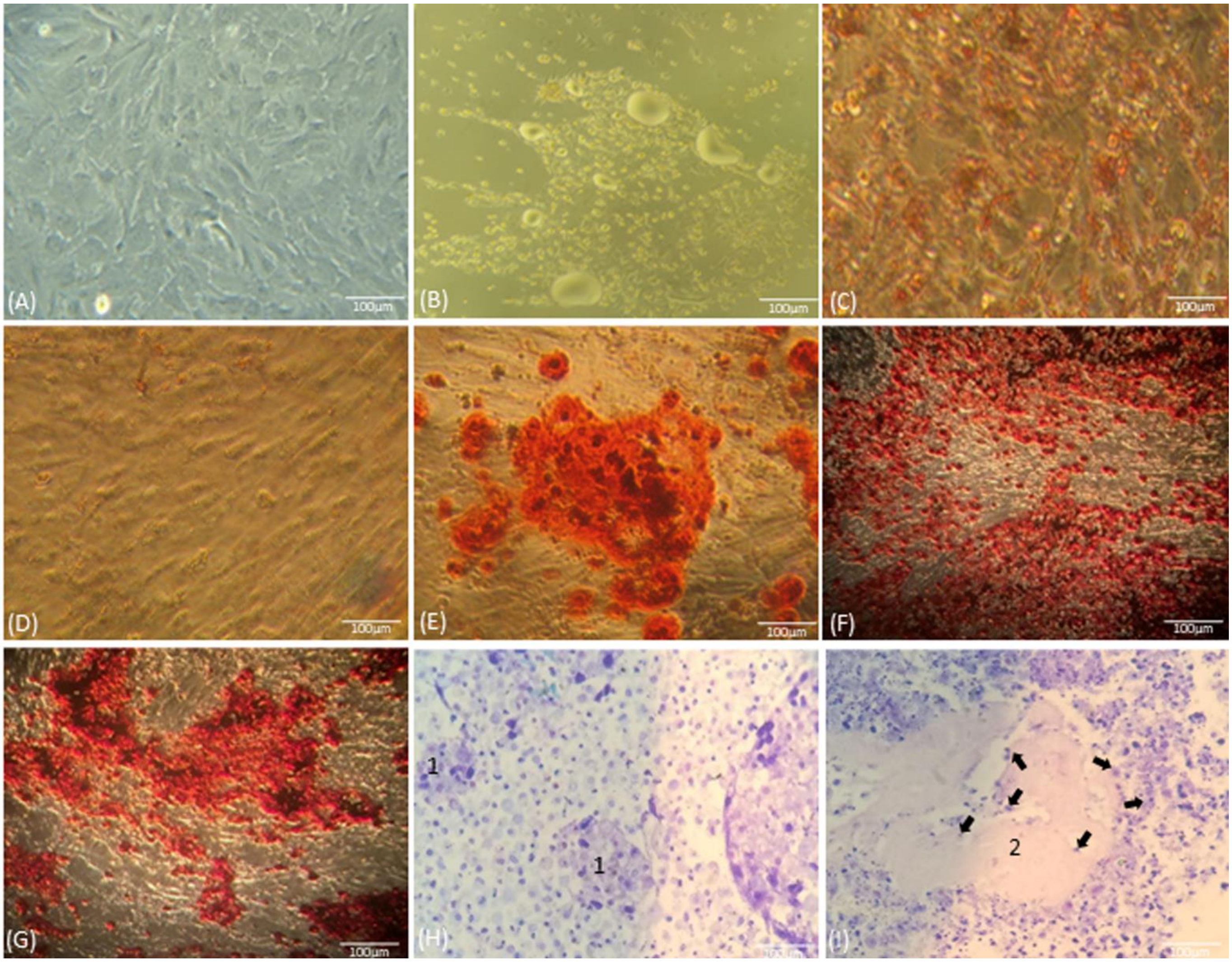

Supplemental Figure S4. Tri-lineage Differentiation. <u>Adipocyte differentiation:</u> **A.** The undifferentiated mesenchymal stem cells can be seen by inverted phase-contrast microscopy; **B.** Detail of adipocytes after MSC differentiation with specific inducing medium, observation by inverted microscopy (not stained); **C.** The appearance of adipocytes after staining with Oil Red®, where lipid droplets with reddish content can be observed; <u>Bone differentiation:</u> **D.** Culture Control, undifferentiated stem cells; **E**. Culture test, the differentiated bone tissue in the presence of an inducing medium. **F, G.** After 14 days, both cultures (control and test) were stained with Alizarin Red®. Red areas indicate a large confluence of calcium deposits, evidence of bone trabeculae. <u>Cartilage differentiation</u>: **H.** Histological section of chondrogenic differentiation from ACS stained with Alcian Blue; 1 – isogenic core. **I.** Arrows correspond to chondrocytes and isogenic nuclei with exuberant extracellular matrix (pink material). 2 = secreting chondrocytes. **Magnification:** 100x.

Supplemental File S4 - Sample processing protocol for SEM

1. After collecting the material, fix it in 2.5% glutaraldehyde in 0.1M phosphate buffer pH 7.3 for at least 4 hours.

Preparation of Monosodium/Disodium Phosphate Buffer - 0.2M pH 7.3

- Solution A: 2.76g of monosodium phosphate (Na_2HPO_4-H_2O), 100 ml of distilled water
- Solution B: 5.66g of dibasic phosphate (Na_2HPO_4-$7H_2O$), 100 ml of distilled water
- OR (depends on the phosphate you have in stock: check the formula on the bottle): 7.17g of dibasic, phosphate (Na_2HPO_4-$12H_2O$), 100 ml of distilled water

To obtain 0.2M sodium phosphate buffer pH 7.3, mix 23 ml of solution A and 77 ml of solution B.

NOTE: To obtain the phosphate buffer at a concentration of 0.1M, simply dilute the volume obtained from the solution to 0.2M with distilled water.

2. Remove specimens from fixative and wash 3 times of 5 minutes each in distilled water.
3. Place the material immersed in 0.5% osmium tetroxide in distilled water for about 30 to 40 minutes.

NOTE: Osmium is a heavy metal. Wear nitrile gloves to handle it. Use just enough osmium to cover the sample.

Preparation of 0.5% osmium tetroxide in distilled water

- Wash the osmium tetroxide ampoule with detergent to remove any possible residue. Soak in water + neutral detergent mixture for about 2 hours.

NOTE: Put the water + detergent in a 250ml beaker, dip the osmium ampoule and use a funnel to keep the ampoule immersed in the solution.

- Break the 1g ampoule of osmium tetroxide into a dark bottle containing 50 ml of distilled water.
- Mix well with a glass rod and break the rest of the ampoule left inside the bottle.
- Makeup to a final volume of 200 ml with distilled water.

4. Wash the material 3 times, 10 minutes each in distilled water.
5. Start dehydration in increasing series of alcohol:
   - 2 times, 10 minutes each in 7.5% alcohol
   - 2 times, 10 minutes each in 15% alcohol
   - 2 times, 10 minutes each in 30% alcohol
   - 2 times, 10 minutes each in 50% alcohol
   - 3 times, 15 minutes each in 70% alcohol
   - 2 times, 15 minutes each in 90% alcohol
   - 2 times, 10 minutes each in 100% alcohol
6. Bring to the critical point.
7. Mount on the “stubs”.
8. Metallize.
